# What Elements of Sport and Exercise Science Should Primary Care Physicians Learn? An Interdisciplinary Discussion

**DOI:** 10.3389/fmed.2021.704403

**Published:** 2021-08-04

**Authors:** Apichai Wattanapisit, Marisa Poomiphak Na Nongkhai, Poramet Hemarachatanon, Soontaraporn Huntula, Areekul Amornsriwatanakul, Chirawat Paratthakonkun, Chirk Jenn Ng

**Affiliations:** ^1^Department of Clinical Medicine, School of Medicine, Walailak University, Nakhon Si Thammarat, Thailand; ^2^Family Medicine Clinic, Walailak University Hospital, Nakhon Si Thammarat, Thailand; ^3^Department of Sport and Exercise Science, School of Medicine, Walailak University, Nakhon Si Thammarat, Thailand; ^4^College of Sports Science and Technology, Mahidol University, Nakhon Pathom, Thailand; ^5^School of Human Sciences (Sport Science, Exercise, and Health), University of Western Australia, Perth, WA, Australia; ^6^Department of Primary Care Medicine, Faculty of Medicine, University of Malaya, Kuala Lumpur, Malaysia

**Keywords:** exercise, physical activity, physician, primary care, sedentary behavior

## Introduction

Primary care physicians (PCPs) contribute to a wide range of healthcare services from health promotion and prevention to treatment and supportive care. A higher PCP-to-population ratio is associated with positive outcomes including longer life expectancy of the population and reduction in cardiovascular, cancer, and respiratory mortality (1). Promoting an active lifestyle is one of the roles of PCPs. Specifically, promoting physical activity (PA) in healthcare settings is an important strategy to reduce the prevalence of insufficient PA and counter excessive sedentary behavior (SB) according to the World Health Organization (WHO) Global Action Plan on Physical Activity 2018–2030 (2). The expected endpoints of promoting PA include a reduction in the risk of premature mortality and several chronic medical conditions such as diabetes mellitus, hypertension, ischemic heart disease, stroke, breast cancer, and colon cancer (3).

The promotion of PA in primary care is an effective intervention for motivating sedentary people to become physically active (4). Several PA and exercise promotion campaigns have been implemented in clinical settings such as Exercise is Medicine (EIM), which emphasizes the assessment of PA of a patient as the fifth vital sign and recommends PA counseling/prescription during clinical consultations (5).

PA promotion is a standard practice in primary care settings. However, the implementation of PA counseling is hindered by several factors such as limited resources (e.g., heavy clinical workload and time constraints); patients' limitations (e.g., physical condition and communication difficulties); and physicians' lack of knowledge and skills and attitude toward PA promotion (6, 7). The last of these is considered as a modifiable factor that can be improved. However, there is little training in PA promotion during undergraduate medical education (8–10) and postgraduate primary care training (11). The continuing professional development training, consisting of face-to-face training, online materials, or workshops, has the potential to fulfill a lack of formal training (12).

The knowledge and skills pertaining to PA promotion in clinical settings cover several aspects of sport and exercise science (13). As PCPs are not specialists in these subject areas, it is important to identify the essential elements for the practices of PCPs. In this article, we present the collective opinion of experts in the field of primary care medicine (AW and CJN), sport and exercise science (MPNN, PH, SH, and CP), and PA (AA) regarding this point.

## Discussions on Sport and Exercise Science

### Defining Physical Activity, Exercise, and Sport

PA is a relatively new concept in many countries and is recognized by the general public as specific activity domains—i.e., sports or exercise. Thus, the term “physical activity” needs to be clarified and distinguished from “exercise” and “sport” to facilitate the promotion of this concept by PCPs.

**Figure 1:**
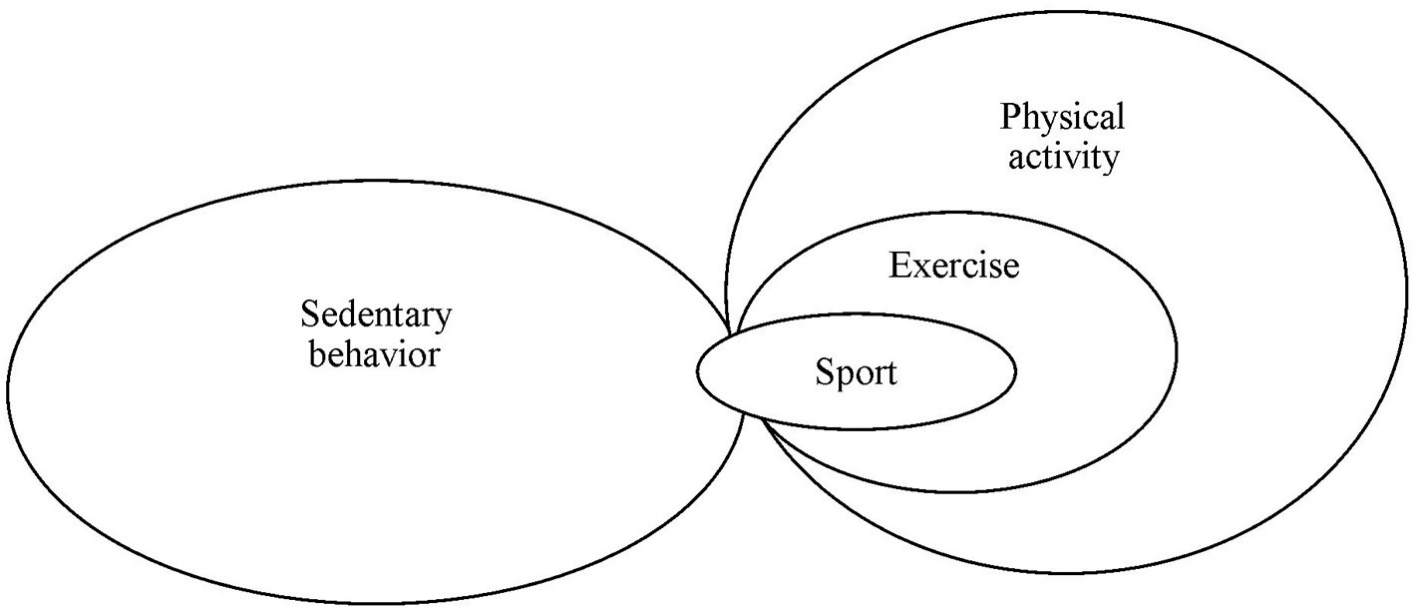
Relationships among sedentary behavior, physical activity, exercise, and sport.

PA is defined as “any bodily movement produced by skeletal muscles that results in energy expenditure” (14). PA incorporates movement activities in daily life. Exercise is planned, structured, repetitive, and is undertaken with the purpose of improving and maintaining physical fitness (14). Exercise is a subset of but not synonymous with PA. However, the two terms are often used interchangeably. Sport is a subset of exercise that consists of a set of rules and defined goal (e.g., competition) (15). Thus, exercise and sport are important domains of PA (Figure 1). For example, running is a PA and can be considered as an exercise. If the running session is a competition, it is defined as sport. Current perceptions and characteristics of sports are variable and dynamic. Some types of sport are not considered as PA or exercise. For example, most eSports require players to control their characters in the virtual world while sitting in front of a monitor during competitions (16). PA is categorized according to its objectives as occupational, transport, domestic, and recreational (including exercise and sport) activities (15), which are collectively known as “PA domains”.

Clarifying the exact meaning of PA is critical for promoting active lifestyles. Given the broad definition of PA, it can be (wrongly) argued that sitting in front of a computer monitor and clicking a mouse with a few fingers—which requires skeletal muscle contraction and uses energy—is PA. However, PA is defined based on the level of energy expenditure. A person engages in both movement and non-movement activities within a 24-h day–night cycle including sleep, SB, and PA. The amount of energy consumed during sleep is approximately 1 metabolic equivalent (1 MET = oxygen uptake of 3.5 ml/kg/min) (17). SB is any waking behavior in a sitting, reclining, or lying posture that expends <1.5 MET of energy (17). PA is characterized by an energy expenditure of ≥1.5 METs while in any posture (17).

Current evidence supports that the benefits of PA are a dose-response basis (18–20). In other words, a higher dose of PA is more beneficial than a lower dose, however, an optimal dose is needed to identify. A very low dose of PA may minimally affect the health benefits while an extremely high dose of PA may cause harm and injuries. The dose of PA depends on two factors—namely, intensity and duration. The intensity of PA can be classified into three levels: light (1.5–2.9 METs), moderate (3–5.9 METs), and vigorous (≥6 METs). Although moderate- to vigorous-intensity PA is considered an effective intensity to improve health, light-intensity PA is also advantageous for health benefits. According to the dose-response basis, it cannot be concluded whether PA, exercise, or sport is the most effective activity. For example, brisk walking at 4 METs (moderate-intensity aerobic PA) for 150 min/week is equal to 600 MET-min/week (about the weekly recommended PA). This dose of running (8 METs for 75 min/week = 600 MET-min/week) may have similar health benefits whether the running session is PA, exercise, or sport. Thus, in this instance, the name given to the activity has no bearing on the benefit that it provides. The challenge for PCPs is designing an appropriate program or intervention for an individual to achieve the recommended levels of PA.

### Integrating Sport and Exercise Science Into Primary Care Medicine

The 2020 WHO guidelines on PA and SB provide recommendations for children and adolescents (aged 5–17 years), adults (aged 18–64 years), older adults (aged ≥65 years), pregnant and postpartum women, and people living with chronic conditions and disabilities (21). The recommendations are simple and can be adopted by both healthy individuals and those with health issues. However, translating public health PA guidelines into clinical practice is challenging. Understanding the concepts of sport and exercise can potentially fill the gaps in the knowledge and skill of PCPs.

Identifying the essential elements of sport and exercise sciences is one of the main objectives of this interdisciplinary discussion. The term “sport” may be omitted from the practices of PCPs because PA or exercise promotion is more relevant to most of the population in primary care. The key competence required in this context is PA counseling or prescription.

Pre-exercise screening and the Frequency, Intensity, Time, and Type (FITT) principle (22) are required knowledge for PCPs to perform PA counseling or prescription. Pre-exercise screening is used to assess an individual's readiness and safety for PA or exercise, while the FITT principle can be adopted as a framework to translate scientific and medical concepts to practice and design a tailored PA program.

### Promoting Safe Physical Activities

PA is generally safe for most people; however, the risks and harms increase with the intensity and dose. PA may increase risks of injuries from minor musculoskeletal injuries to sudden cardiac arrest. One study shows that a PA-related injury over 30 days during walking, gardening, weightlifting, outdoor bicycling, and performing aerobics is 0.9 to 2.4% (23). The incidence of sudden cardiac arrest related to exercise is still low (0.6 to 2.1 per 100,000 person-years) compared with out-of-hospital cardiac arrest (65.4 to 108.9 per 100,000 person-years) (22). There are several challenges faced by PCPs when providing care and PA/exercise prescriptions to patients; the two main ones are discussed in detail below.

Firstly, readiness and safety are critical issues for patients engaging in PA. Health screening may be required to ensure readiness and thereby reduce the risks of adverse events. A variety of approaches for this purpose can be adopted in clinical practice (22). For example, self-screening tools such as Physical Activity Readiness for Everyone (PAR-Q+) and the electronic Physical Activity Readiness Medical Examination (ePARmed-X+) are freely available online for public access (https://eparmedx.com/). For patients who require medical advice, PCPs can perform an evaluation based on patients' current PA levels, history of illness as well as signs and symptoms prior to their engagement in PA according to the American College of Sports Medicine's health screening process (24). High-risk patients should be given careful consideration and may need to be referred to medical specialists prior to initiation of PA.

Secondly, the depth of disease-specific knowledge required by PCPs to promote PA is a major concern. Sport and exercise scientists are content experts with detailed knowledge of exercise physiology, biomechanics, and anatomy and possess tools to evaluate patients with chronic conditions. It can be an exhausting task for PCPs to study all of these sub-disciplines of sport and exercise science along with disease-specific exercise guidelines (e.g., for cancer survivors or patients with heart disease or musculoskeletal disorders). Therefore, PCPs only need to understand the basic concepts of PA or exercise and the contraindications and precautions for PA for specific medical conditions. A scheme for referrals to sport and exercise specialists is strongly suggested for patients who require special care.

### The FITT Principle as a Framework to Promote Physical Activity

PA recommendations include specific details for each group within the population. For example, the recommendation for healthy adults (18–64 years) is 150–300 min/week of moderate-intensity or 75–150 min/week of vigorous-intensity aerobic PA (21). Additionally, adults should undertake muscle-strengthening activities at moderate or greater intensity ≥2 days/week (21). The time spent engaging in SB should be limited and replaced with PA at any intensity, including light activities (21).

Notably, PA recommendations for adults include details on the frequency, intensity (i.e., light to vigorous), time (i.e., 150–300 min/week), and type (aerobic). PCPs can rely on the FITT principle based on WHO PA recommendations when providing PA counseling. The recommended 150–300 min/week of moderate-intensity PA can be designed in different ways. The 150 min/week target can be achieved with five 30-min sessions per week of brisk walking. Alternatively, the target can be achieved by combining 120 min of recreational swimming and 30 min of gardening with heavy tools.

Vague advice such as “move more and sit less”, “go exercise”, or “increase your PA”, are superficial and may be ineffective in changing people's behaviors. The FITT principle can provide a supportive framework that can aid PCPs in PA counseling or in writing a PA/exercise prescription. Otherwise, general advice regarding SB—e.g., “limit sitting time” or “reduce time spent on SB”—may be suitable. The informative content of PA counseling provided by PCPs can help individuals to achieve the recommended PA goals.

## Conclusion

This interdisciplinary discussion highlights the important role of PCPs in promoting PA and reducing SB in the primary care setting. The basic concepts of sport and exercise sciences, as well as WHO PA recommendations, constitute essential knowledge for PCPs that should be integrated into their professional practices. Pre-PA/exercise screening may be required, particularly for high-risk patients. Safety issues must also be taken into account when providing PA/exercise counseling or prescriptions. The FITT principle is suggested as a framework to perform the PA/exercise counseling and prescription. Integration of sport and exercise science into the primary care practice requires an understanding and incorporation of underlying concepts, as well as coordination between PCPs and sport/exercise scientists.

## Author Contributions

AW and MPNN conceived the manuscript. PH, MPNN, SH, AA, CP, and CJN contributed comments. AW wrote the first draft of the manuscript. AW and AA edited the manuscript. All authors participated in the discussions, read and approved the final version of the manuscript, and agreed with the order of presentation of the authors.

## Conflict of Interest

The authors declare that the research was conducted in the absence of any commercial or financial relationships that could be construed as a potential conflict of interest.

## Publisher's Note

All claims expressed in this article are solely those of the authors and do not necessarily represent those of their affiliated organizations, or those of the publisher, the editors and the reviewers. Any product that may be evaluated in this article, or claim that may be made by its manufacturer, is not guaranteed or endorsed by the publisher.

## References

[1] Basu S Berkowitz SA Phillips RL Bitton A Landon BE Phillips RS. Association of primary care physician supply with population mortality in the United States, 2005-2015. JAMA Intern Med. (2019) 179:506–14. 10.1001/jamainternmed.2018.7624 30776056 PMC6450307

[2] World Health Organization. Global Action Plan on Physical Activity 2018–2030: More Active People for a Healthier World. Geneva, Switzerland: World Health Organization (2018).

[3] Warburton DER Bredin SSD. Health benefits of physical activity: a systematic review of current systematic reviews. Curr Opin Cardiol. (2017) 32:541–56. 10.1097/HCO.0000000000000437 28708630

[4] Orrow G Kinmonth A-L Sanderson S Sutton S. Effectiveness of physical activity promotion based in primary care: systematic review and meta-analysis of randomised controlled trials. BMJ. (2012) 344:e1389. 10.1136/bmj.e1389 22451477 PMC3312793

[5] Bowen PG Mankowski RT Harper SA Buford TW. Exercise is medicine as a vital sign: challenges and opportunities. Transl J Am Coll Sports Med. (2019) 4:1–7. 30828640 PMC6392189

[6] Hébert ET Caughy MO Shuval K. Primary care providers' perceptions of physical activity counselling in a clinical setting: a systematic review. Br J Sports Med. (2012) 46:625–31. 10.1136/bjsports-2011-090734 22711796

[7] Wattanapisit A Thanamee S Wongsiri S. Physical activity counselling among GPs: a qualitative study from Thailand. BMC Fam Pract. (2019) 20:72. 10.1186/s12875-019-0968-x 31142277 PMC6540406

[8] Wattanapisit A Vijitpongjinda S Saengow U Amaek W Thanamee S Petchuay P. Results from the Medical School Physical Activity Report Card (MSPARC) for a Thai medical school: a mixed methods study. BMC Med Educ. (2018) 18:288. 10.1186/s12909-018-1408-7 30514276 PMC6278075

[9] Stoutenberg M Stasi S Stamatakis E Danek D Dufour T Trilk JL. Physical activity training in US medical schools: preparing future physicians to engage in primary prevention. Phys Sportsmed. (2015) 43:388–94. 10.1080/00913847.2015.1084868 26365470

[10] Strong A Stoutenberg M Hobson-Powell A Hargreaves M Beeler H Stamatakis E. An evaluation of physical activity training in Australian medical school curricula. J Sci Med Sport. (2017) 20:534–8. 10.1016/j.jsams.2016.10.011 28209318

[11] Wattanapisit A Tuangratananon T Thanamee S. Physical activity counseling in primary care and family medicine residency training: a systematic review. BMC Med Educ. (2018) 18:159. 10.1186/s12909-018-1268-1 29970092 PMC6029015

[12] Brannan M Bernardotto M Clarke N Varney J. Moving healthcare professionals - a whole system approach to embed physical activity in clinical practice. BMC Med Educ. (2019) 19:84. 10.1186/s12909-019-1517-y 30876426 PMC6419815

[13] Wattanapisit A Petchuay P Wattanapisit S. Developing a training programme in physical activity counselling for undergraduate medical curricula: a nationwide Delphi study. BMJ Open. (2019) 9:e030425. 10.1136/bmjopen-2019-030425 31481372 PMC6731937

[14] Caspersen CJ Powell KE Christenson GM. Physical activity, exercise, and physical fitness: definitions and distinctions for health-related research. Public Health Rep. (1985) 100:126–31. 3920711 PMC1424733

[15] Khan KM Thompson AM Blair SN Sallis JF Powell KE Bull FC. Sport and exercise as contributors to the health of nations. Lancet. (2012) 380:59–64. 10.1016/S0140-6736(12)60865-4 22770457

[16] Wattanapisit A Wattanapisit S Wongsiri S. Public health perspectives on eSports. Public Health Rep. (2020) 135:295–98. 10.1177/0033354920912718 32237971 PMC7238713

[17] Tremblay MS Aubert S Barnes JD. Sedentary behavior research network (SBRN) - terminology consensus project process and outcome. Int J Behav Nutr Phys Act. (2017) 14:75. 10.1186/s12966-017-0525-8 28599680 PMC5466781

[18] Geidl W Schlesinger S Mino E Miranda L Pfeifer K. Dose–response relationship between physical activity and mortality in adults with noncommunicable diseases: a systematic review and meta-analysis of prospective observational studies. Int J Behav Nutr Phys Act. (2020) 17:109. 10.1186/s12966-020-01007-5 32843054 PMC7448980

[19] Ekelund U Tarp J Steene-Johannessen J Hansen BH Jefferis B Fagerland MW. Dose-response associations between accelerometry measured physical activity and sedentary time and all cause mortality: systematic review and harmonised meta-analysis. BMJ. (2019) 366:l4570. 10.1136/bmj.l4570 31434697 PMC6699591

[20] Kyu HH Bachman VF Alexander LT Mumford JE Afshin A Estep K. Physical activity and risk of breast cancer, colon cancer, diabetes, ischemic heart disease, and ischemic stroke events: systematic review and dose-response meta-analysis for the Global Burden of Disease Study 2013. BMJ. (2016) 354:i3857. 10.1136/bmj.i3857 27510511 PMC4979358

[21] Bull FC Al-Ansari SS Biddle S Borodulin K Buman MP Cardon G. World Health Organization 2020 guidelines on physical activity and sedentary behaviour. Br J Sports Med. (2020) 54:1451–62. 10.1136/bjsports-2020-102955 33239350 PMC7719906

[22] Wattanapisit A Wattanapisit S Wongsiri S. Overview of physical activity counseling in primary care. Korean J Fam Med. (2020). 10.4082/kjfm.19.0113 32429011 PMC8321902

[23] Powell KE Heath GW Kresnow M-J Sacks JJ Branche CM. Injury rates from walking, gardening, weightlifting, outdoor bicycling, and aerobics. Med Sci Sports Exerc. (1998) 30:1246–9. 10.1097/00005768-199808000-00010 9710864

[24] Riebe D Franklin BA Thompson PD Garber CE Whitfield GP Magal M. Updating ACSM's recommendations for exercise preparticipation health screening. Med Sci Sports Exerc. (2015) 47:2473–9. 10.1249/MSS.0000000000000664 26473759

